# Photoactivation of lysosomally sequestered sunitinib after angiostatic treatment causes vascular occlusion and enhances tumor growth inhibition

**DOI:** 10.1038/cddis.2015.4

**Published:** 2015-02-12

**Authors:** P Nowak-Sliwinska, A Weiss, J R van Beijnum, T J Wong, W W Kilarski, G Szewczyk, H M W Verheul, T Sarna, H van den Bergh, A W Griffioen

**Affiliations:** 1 Institute of Chemical Sciences and Engineering, Swiss Federal Institute of Technology (EPFL), Lausanne, Switzerland; 2 Department of Medical Oncology, Angiogenesis Laboratory, VU University Medical Center, Amsterdam, The Netherlands; 3 Institute of Bioengineering, School of Life Sciences, SV IBI LLCB, Swiss Federal Institute of Technology (EPFL), Lausanne, Switzerland; 4 Department of Biophysics, Faculty of Biochemistry, Biophysics and Biotechnology, Jagiellonian University, Krakow, Poland

## Abstract

The angiogenesis inhibitor sunitinib is a tyrosine kinase inhibitor that acts mainly on the VEGF and PDGF pathways. We have previously shown that sunitinib is sequestered in the lysosomes of exposed tumor and endothelial cells. This phenomenon is part of the drug-induced resistance observed in the clinic. Here, we demonstrate that when exposed to light, sequestered sunitinib causes immediate destruction of the lysosomes, resulting in the release of sunitinib and cell death. We hypothesized that this photoactivation of sunitinib could be used as a vaso-occlusive vascular-targeting approach to treating cancer. Spectral properties of sunitinib and its lysosomal accumulation were measured *in vitro*. The human A2780 ovarian carcinoma transplanted onto the chicken chorioallantoic membrane (CAM) and the Colo-26 colorectal carcinoma model in Balb/c mice were used to test the effects of administrating sunitinib and subsequently exposing tumor tissue to light. Tumors were subsequently resected and subject to immunohistochemical analysis. In A2780 ovarian carcinoma tumors, treatment with sunitinib+light resulted in immediate specific angio-occlusion, leading to a necrotic tumor mass 24 h after treatment. Tumor growth was inhibited by 70% as compared with the control group (**P<0.0001). Similar observations were made in the Colo-26 colorectal carcinoma, where light exposure of the sunitinib-treated mice inhibited tumor growth by 50% as compared with the control and by 25% as compared with sunitinib-only-treated tumors (*N*≥4; *P*=0.0002). Histology revealed that photoactivation of sunitinib resulted in a change in tumor vessel architecture. The current results suggest that the spectral properties of sunitinib can be exploited for application against certain cancer indications.

Angiogenesis inhibitors are currently firmly implemented in the clinical management of cancer. For example, sunitinib has been approved for the treatment of advanced renal cell carcinoma (RCC),^1^ gastrointestinal stromal tumors (GIST)^2^ and pancreatic neuroendocrine tumors.^3^ Studies assessing its activity against other tumor types are currently underway.^4^ Sunitinib was developed as an angiogenesis inhibitor^5^ and revolutionized the management of advanced RCC and GIST. Its mode of action is based on the suppression of the tyrosine kinase activity of several growth factor receptors, mainly VEGFR2 and PDGFR (alpha and beta).^6^ It has been previously shown that sunitinib is sequestered by tumor cells in their lysosomal compartments.^7^ This sequestration, which limits the exposure of other parts of the cell to sunitinib, is part of a drug-induced resistance mechanism that has also been clinically observed.^7^ The sequestration and accumulation of sunitinib in the lysosomes is similar to a phenomenon that has been described for certain other chemotherapeutics^8, 9^ or photosensitizing compounds,^10^ and depends, at least to some extent, on the hydrophobic and weak basic features of the molecule. We discovered a particular characteristic of sunitinib based on its optical properties, which may be helpful in the treatment of certain cancers. On the basis of the spectral features of sunitinib, we hypothesized that the drug may have photosensitizer-like properties.^11^ If so, exposure to light of an appropriate wavelength could lead to the disruption of the lysosomal membrane, the release of sunitinib and re-exposure of the cell to this molecule. This could lead to further cell damage and eventually cell death. In this study, we show that the exposure of sunitinib-treated cells to light of an appropriate wavelength excites the molecule, which leads to the generation of reactive oxygen species (ROS). We show in two tumor models, that is, human ovarian carcinoma (A2780) xenografted on the chorioallantoic membrane of the chicken embryo (chicken chorioallantoic membrane (CAM)) and in colorectal carcinoma in Balb/c mice, that treatment with sunitinib and its subsequent photoexcitation leads to significant occlusion of the vasculature and inhibition of tumor growth. Our results indicate that additional antitumor activity can be obtained after the normal use of sunitinib through its use as a photosensitizer and the application of light. The combination of classical sunitinib-induced angiostasis with the re-exposure of tumor cells to sunitinib after the destruction of lysosomes and photodynamic vessel obstruction can lead to a strategy that may be applicable in patients.

## Results

### Spectral properties of sunitinib and its sequestration in endothelial cell lysosomes

**Figure 1:**
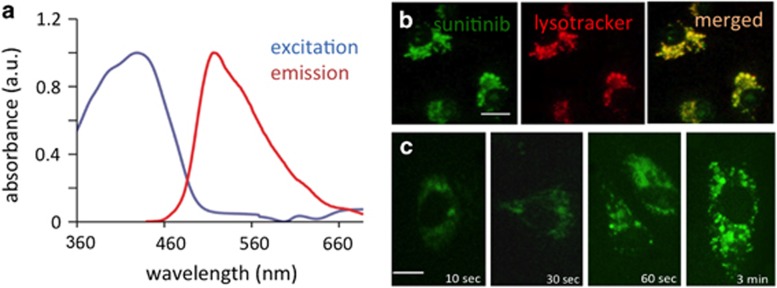
Spectral properties of sunitinib and lysosomal accumulation in endothelial cells. (**a**) Normalized absorption and emission spectra of sunitinib in 0.1% DMSO in 0.9% NaCl. (**b**) Colocalization of sunitinib (green) and lysotracker (red) in cultured human umbilical vein endothelial cells (HUVEC). Bar in left panel represents 10 *μ*m. (**c**) Rapid uptake and lysosomal accumulation of sunitinib in HUVEC. Bar in left panel represents 5 *μ*m

Absorption and emission spectra of sunitinib were generated in an aqueous solution (Figure 1a). Sunitinib light absorbance was found to be in a broad range of 340–480 nm, with the absorption maximum at 429 nm. Sunitinib showed strong fluorescence with a maximum at 540 nm. Fluorescence microscopy revealed that incubating human umbilical vein endothelial cells (HUVEC) in 1 *μ*M sunitinib for an hour resulted in the sequestration of sunitinib in subcellular compartments. Sunitinib, being a hydrophobic weak base (acid dissociation constant of 8.95),^7^ was predominantly localized in the lysosomes of the cells. A near-100% colocalization of sunitinib and lysotracker fluorescence supported this observation (Figure 1b). Uptake of sunitinib in endothelial cells was observed as early as 10 s after exposure, with visible accumulation in lysosomes after 30 s (Figure 1c).

### Light exposure of endothelial cells with sequestered sunitinib induces cell cytotoxicity

**Figure 2:**
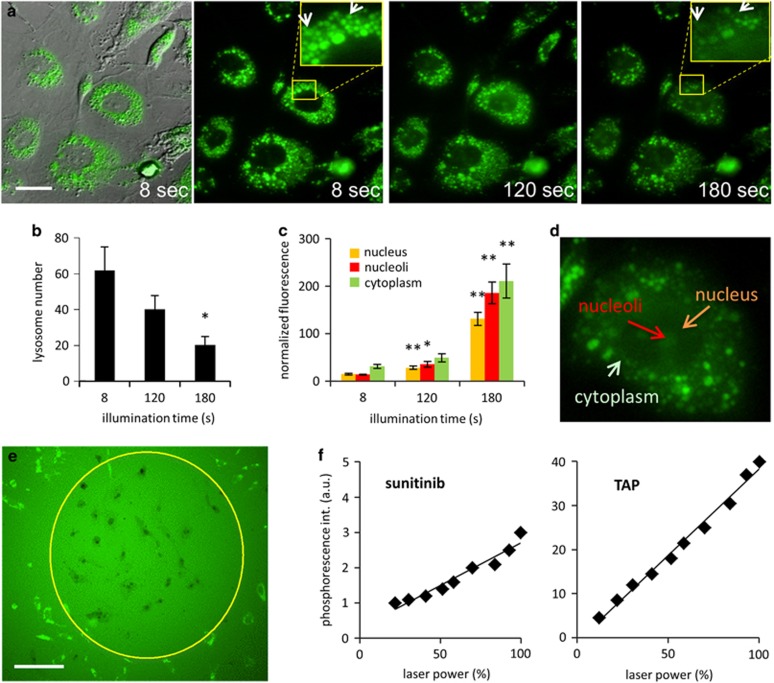
Photoactivation of lysosomal sunitinib causes endothelial cell death. (**a**) Cellular localization of sunitinib in endothelial cells, after 1 h of exposure to 1 *μ*M sunitinib, visualized by differential interference contrast and fluorescence microscopy (left panel, bar represents 5 *μ*m) at different time points. Arrows indicate ruptured lysosomes. (**b**) Quantification of lysosomes (*N*=4 cells). (**c** and **d**) Gradual increase in cytoplasmic and nuclear and nucleoli-associated fluorescence. Values are normalized to background fluorescence at each time point to account for photobleaching (*N*=4 cells). (**e**) Trypan blue staining of HUVEC cells incubated for 1 h in 1 *μ*M sunitinib and irradiated with light (*λ*_ex_=420 nm) for 60 min. Bar represents 50 *μ*m. (**f**) Singlet oxygen quantum yield of sunitinib, measured by photoexcitation and detection of phosphorescence at 355 nm. TAP was used as a positive control. *P=0.02; **P<0.008

HUVEC cells were incubated with sunitinib (1 *μ*M) for 1 h at 37 °C, to allow sufficient amounts of sunitinib being sequestered in the lysosomes. Cells were then washed with drug-free medium and subjected to illumination for a time period of 5 min under a fluorescence microscope with excitation wavelengths of 420±20 nm and emission wavelengths of >470 nm. Upon light illumination, a rapid destruction and disappearance of sunitinib-containing lysosomes was observed (200 mW/cm^2^, Figure 2a). Over 3 min of light exposure, >70% of the sunitinib-containing lysosomes disappeared (Figure 2a, quantification Figure 2b), whereas >90% of lysosomes were destroyed after 5 min (a movie of this experiment can be viewed at the secured site: www.angiogenesis.nl/NowakSliwinska%20submission.htm). The destruction of lysosomes, visible as multiple asynchronous flashes, was accompanied by a gradual increase in cytoplasmic and nuclear fluorescence (*λ*_em_=405 nm). After 180 s of light exposure, nuclear fluorescence increased over sevenfold, whereas the cytoplasmic fluorescence increased over 13-fold (images in Figure 2a, quantification in Figure 2c). Nuclear localization of sunitinib was further confirmed using confocal microscopy (Supplementary Figure 1A). Quantification of the sunitinib in a z-stack of images (Supplementary Figure 1A) showed higher sunitinib accumulation in the nuclei of cells as compared with the cytoplasm (Supplementary Figure 1B). Interestingly, within the nucleus, sunitinib appeared to accumulate preferentially in or associated to the nucleoli (Figures 2c and d). A subsequent trypan blue exclusion assay revealed that the light-exposed cells were dead (Figure 2e), whereas cells exposed to sunitinib or light only (Supplementary Figure 2) were still capable of dye exclusion.

The absorption spectrum of sunitinib before and after light exposure showed no significant changes in the absorbance peaks, suggesting that light exposure did not lead to structural changes or damage of sunitinib molecules (Supplementary Figure 3), a result that was similar for the clinically approved photosensitizer Visudyne (Novartis Pharma Inc., Hettlingen, Switzerland). These results suggest that sunitinib has photosensitizer-like activity, such as light-induced generation of ROS. To verify this, we measured the singlet oxygen generation (type-II photochemistry) of sunitinib by direct detection of its phosphorescence at 1270 nm, after pulsed excitation with 355 nm laser radiation in acetonitrile and DMSO (Sigma-Aldrich, Buchs, Switzerland). The quantum yield of singlet oxygen formation was 5–7% of the quantum yield of tetraanilinoporphyrin (TAP; absolute yield of singlet oxygen generation is 0.7), used as a positive control (Figure 2f). It should be noted that the excitation wavelength of 355 nm is well below the excitation peak of sunitinib at 429 nm. Therefore, in this experiment the induction of singlet oxygen may be an underrepresentation of the real effect.

Measurement of other ROS using EPR-spin trapping and DMPO as a spin trap^12, 13^ revealed no induction of ROS. More information is in the Supplementary Material. The results suggest that sunitinib-dependent lysosomal photodestruction is mediated, at least in part, by the generation of singlet oxygen, leading to the rapid rupture of the lysosomal membrane, resulting in the release of sunitinib into the cytoplasm and nucleus, leading to toxicity and cell death.

### Photoexcitation of accumulated sunitinib causes vascular occlusion in the CAM

**Figure 3:**
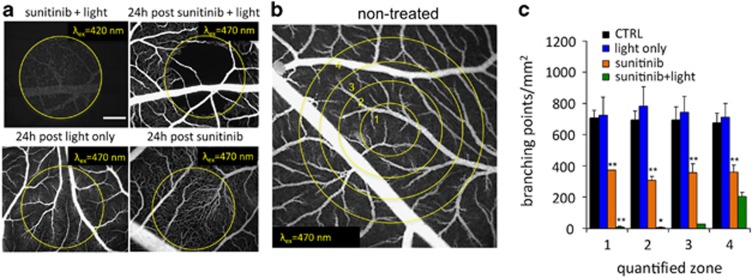
Vascular effects after photoactivation of sunitinib observed in the CAM model. (**a**) Sunitinib fluorescence after i.v. administration (12 *μ*g/embryo, 1 min prior to light exposure, *λ*_ex_=420±20 nm, 270 mW/cm^2^). Representative FITC-dextran fluorescence angiography (12 *μ*g/embryo, 20 kDa, *λ*_ex_=470 nm, *λ*_em_=520 nm) before (left image, upper row), 24 h after sunitinib and light treatment (right image, upper row), 24 h post light-only treatment (left image, bottom row) and 24 h post administration of sunitinib only (12 *μ*g/embryo, right image, bottom row). Bar represents 500 *μ*m. (**b**) Fluorescence angiogram of non-treated CAM vasculature at embryo development day 13. Circles indicate the four quantification zones used in image processing. (**c**) Quantification of branching points/mm^2^ for areas 1–4, as shown in **c**. Results are expressed as means±S.E.M., *N*=2–4. Sunitinib alone resulted in a moderate but significant reduction of branching points in all quantified zones (**P<0.01, *t*-test). Sunitinib+light significantly reduced the number of branching points in zone 1 (**P=1.7E−07, *t*-test) and zone 2 (*P=0.0039, *t*-test) as compared with sunitinib alone

The CAM model was used to check for a vascular component in photoactivation of sunitinib. Sunitinib (12 *μ*g/embryo) was administered i.v. 1 min prior to light exposure (sunitinib excitation *λ*_ex_=420±20 nm was performed with a light fluence of 130 J/cm^2^ and irradiance of 270 mW/cm^2^; sunitinib fluorescence was detected with *λ*_em_>520 nm; Figure 3a, first image). One minute after its i.v. injection, sunitinib is already beginning to accumulate in the endothelium (data not shown). The light activation of sunitinib at the applied conditions resulted in complete capillary bed occlusion within the treated area, which was still visible 24 h after treatment, as shown in the fluorescein isothiocyanate (FITC)-dextran angiography (*λ*_ex_=420±20 nm, *λ*_em_>520 nm, Figure 3a, top right image). Quantification of the vascular changes was preformed based on image analysis within four concentric circles (within the illumination zone for light-treated samples, see Figure 3b), and is presented as a number of branching points per mm^2^. The analysis revealed a 98% reduction of blood vessels in photo-exposed areas 1 (**P=0.0015) and 2 (**P=0.0015), 95% in area 3 (**P=0.0017, *t*-test), and an approximate 70% reduction in area 4 (**P=0.008, Figure 3c) for sunitinib+light *versus* light only. Application of light alone did not have any vaso-occlusive effect. Sunitinib alone resulted in a moderate but significant reduction of branching points in all quantified zones (**P<0.01). Sunitinib+light significantly reduced the number of branching points in zone 1 (**P=1.7E−07) and zone 2 (*P=0.0039) as compared with sunitinib alone.

The angio-occlusive potential of photoactivated sunitinib was also assessed with a lower dose of sunitinib and a lower light fluence. An i.v. dose of only 2 ng/embryo, 1 min prior to low-dose light exposure (*λ*_ex_=420±20 nm, 34 J/cm^2^, 70 mW/cm^2^) already caused a significant vaso-occlusion in 60–70% of the capillaries (Supplementary Figure 4).

### Photoactivation of sunitinib leads to tumor growth inhibition of A2780 human ovarian carcinoma grown on the CAM

**Figure 4:**
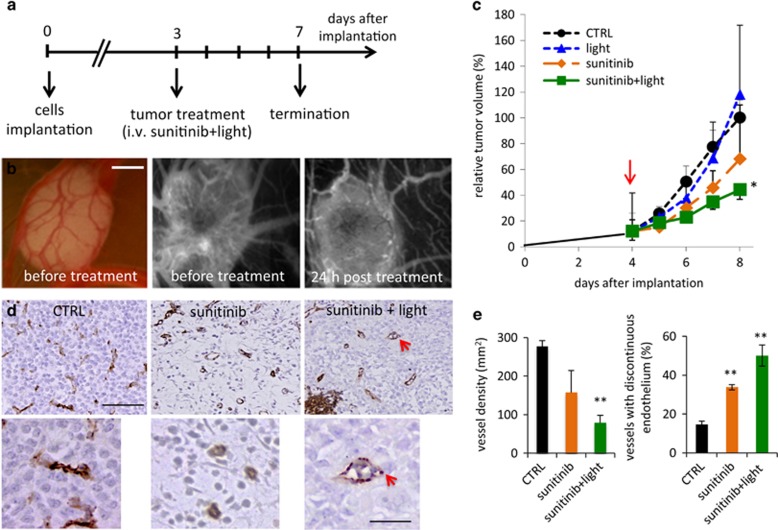
Photoinduced cytotoxicity of sunitinib in A2780 ovarian carcinoma grown on the CAM. (**a**) Experimental design. (**b**) Bright-field image of A2780 tumors grown on the CAM (left image). FITC-dextran angiograms of the sunitinib-treated A2780 tumors prior to light exposure (middle image) and 24 h post light exposure (right image). Bar represents 1 mm. (**c**) Relative tumor growth curves of control tumors (CTRL, treated with 0.1% DMSO) and tumors treated with sunitinib on day 3, with or without subsequent light exposure. Tumor volume was normalized by the respective tumor volume on the first day of treatment and represented as the mean tumor volume±S.E.M. A two-way ANOVA test was performed *versus* CTRL (*N*=14) with the following results: sunitinib+light (**P<0.0001, *N*=12), sunitinib (*P=0.026, *N*=3) and light alone (not significant, *N*=2). (**d**) Representative images of immunohistochemical staining for CD31 of tumors resected on the last day of the experiment. Red arrow indicates vessel with discontinuous endothelial lining. (**e**) Quantification of vessel densities (per mm^2^) and the percentage of blood vessels with discontinuous vessel endothelium in the CTRL, sunitinib+light and sunitinib-treatment groups; results are expressed as means±S.E.M. **P<0.01, *N*=3–5 images

Human A2780 ovarian carcinoma spheroids were transplanted onto the CAM and treated as presented in Figure 4a. Vascularized tumors were detected 3 days post implantation (Figure 4b, left and middle panels). Tumors were divided into four treatment groups: (i) control, (ii) light, (iii) sunitinib, or (iv) sunitinib+light. Treatment was given once at day 3. Twenty-four hours post treatment with sunitinib+light, the tumor vasculature was majorly unperfused, as visualized by fluorescence angiography (Figure 4b, right panel). The growth of tumors treated with sunitinib+light (group iv) was inhibited by ~70% (**P<0.0001, ANOVA, *N*=12) *versus* control tumors (group i) (Figure 4c). Sunitinib administered alone inhibited growth by 32% (*P=0.026, *N*=3). Immunohistochemical staining showed areas of tumor destruction and necrosis-associated hemorrhages in the tumors treated with sunitinib+light (Figure 4d, right image). Quantification of CD31 staining (Figure 4e) revealed reduced vessel density in the sunitinib and the sunitinib+light groups, reaching only significance in the latter group (**P<0.01). Vascular disruption, observed by a discontinuous endothelial lining, was significantly increased in the sunitinib+light group, as compared with the control and sunitinib-treated groups (**P<0.01 *versus* control, *P=0.04 for sunitinib *versus* sunitinib+light; Figure 4d, bottom images, and quantification in Figure 4e).

### Photoactivation of sunitinib inhibits tumor growth in a colon carcinoma mouse model

**Figure 5:**
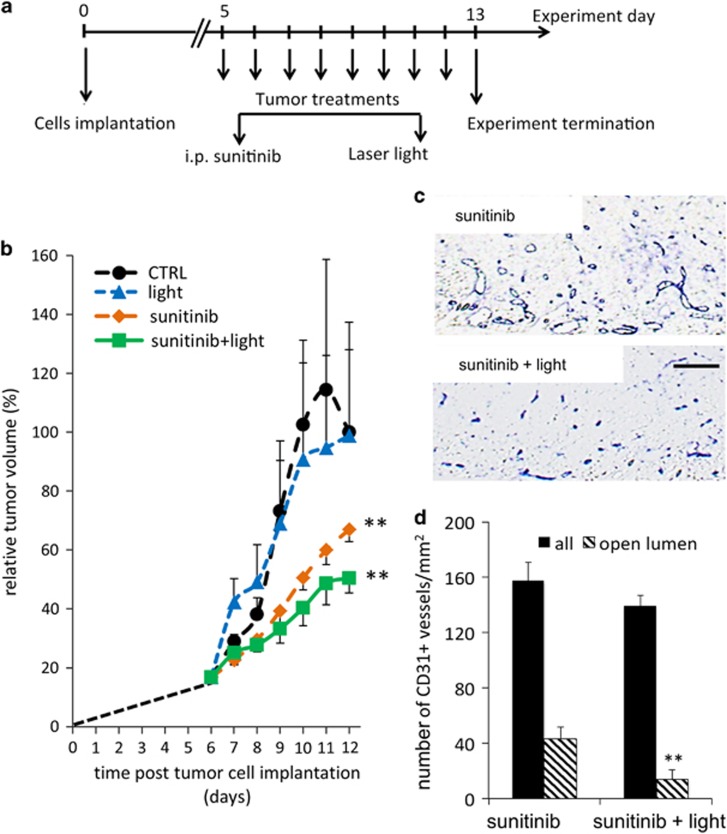
Inhibition of tumor growth in Colo-26 murine colon carcinoma tumors. (**a**) Experimental design. (**b**) Tumor growth curves of the CTRL, light-only, sunitinib-only and sunitinib+light groups. Tumor growth was significantly inhibited by treatment with sunitinib alone (**P<0.0001) *versus* CTRL. Light therapy did not have any effect on tumor growth (*P*=0.95 *versus* CTRL), whereas the combination of sunitinib and light resulted in a significant enhancement of tumor growth inhibition (**P<0.0002 *versus* sunitinib only, ANOVA), *N*≥4. Results are expressed as means±S.E.M. (**c**) Immunohistochemical staining for blood vessels with CD31 antibody. (**d**) Quantification of microvessel density and the number of vessels with an open lumen. Results are expressed as means±S.E.M.

Balb/c mice were inoculated on the flanks with murine Colo-26 colorectal carcinoma cells. Four experimental groups were executed: controls, sunitinib only (i.p. 40 mg/kg daily), light only (fluence of 100 J/cm^2^ and a delivered irradiance of 100 mW/cm^2^) and sunitinib+light. When tumor size reached approximately 5–6 mm in diameter (day 5 after inoculation), treatment of the mice was started (for 8 days, see Figure 5a). Light was always applied 3 h after sunitinib administration. Tumor growth was monitored every day and mice were killed on day 9 and tumors were resected. Tumor growth was significantly inhibited by 33% when treated with sunitinib alone (**P<0.0001) *versus* CTRL. Although light therapy alone did not have any effect on tumor growth (*P*=0.95 *versus* CTRL), the combination of sunitinib and light resulted in a significant enhancement of tumor growth inhibition (**P=0.0002 *versus* sunitinib only, Figure 5b). Quantification of microvessel density in the superficial area of treated tumors (Figure 5c) revealed a slight but not significant decrease in the number of blood vessels (Figure 5d). However, the architecture of the vessels in both groups was found to be different with a significant decrease in the number of vessels with an open lumen (*P<0.02) in the sunitinib+light-treated tumors (Figure 5d). This suggests that the combined treatment resulted in obstruction of the blood flow and tissue damage.

## Discussion

We have previously shown that sunitinib is taken up by tumor cells, where it is sequestered and accumulated in the lysosomal compartment.^7^ This sequestration has a role in protection against sunitinib toxicity and more importantly, in the development of resistance against sunitinib.

Here we show that sunitinib is also sequestered in the lysosomes of endothelial cells *in vitro* and in the endothelium of tumor microvasculature. The photoactivation of sequestered sunitinib *in vitro* resulted in almost immediate rupture of the lysosomes, seen as ‘exploding' vesicles (see Supplementary Video at www.angiogenesis.nl/NowakSliwinska%20submission.htm), followed by release of sunitinib into the cytosol and the nucleus, resulting in additional cytotoxic effects. The same phenomenon applied *in vivo* on the vasculature of the CAM model resulted in photoinduced vascular obstruction, similar to that seen with clinically used photosensitizers during vascular-targeted photodynamic therapy, such as Visudyne.^14^ Exposure of sunitinib-treated tumors to an appropriate wavelength of light significantly enhanced tumor growth inhibition in two separate preclinical tumor xenograft models for ovarian and colorectal carcinomas. Ovarian carcinoma tumors grown on the CAM provide an ideal, easily accessible model to examine the effects of photoactivated sunitinib *in vivo*, because the small superficial tumor allowed for direct light irradiation compensating for limited light penetration depth in tissue at this wavelength. As it was demonstrated that sunitinib accumulates in both tumor^7^ and endothelial cells, the observed effect after exposing the tumors of sunitinib-treated animals to light is the resultant effect on damage to both tumor and endothelial cell compartments.

**Figure 6:**
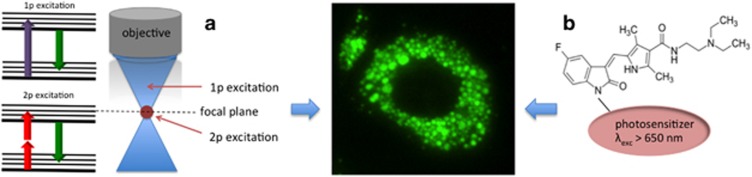
Schematic representation of how to ameliorate sunitinib photoactivation to increase the therapeutic benefit and overcome resistance. This can be done by (**a**) using two-photon (2p) excitation and/or (**b**) conjugation of sunitinib to a photosensitizer with long-wave excitation spectrum

For most current clinical applications, however, the peak excitation wavelength of sunitinib at 420 nm remains problematic. First, this excitation spectrum largely overlaps with the absorption spectrum of hemoglobin.^15^ Thus, in well-perfused tissues, hemoglobin will absorb a large part of the light energy. For application in vascularized tissues there are, however, suboptimal excitation wavelengths, outside the spectrum of hemoglobin that would still specifically excite sunitinib. In addition, the excitation wavelength of 420 nm has a limited tissue penetration depth of ~2 mm, depending on the tissue. Modern photosensitizers, such as the chlorine- or porphyrin-type compounds, have excitation wavelengths in the near-infrared part of the spectrum, where penetration depth is significantly higher for the red light than for blue light.^16^ This limits the possible application of such a treatment for cancer to superficial lesions. However, the activation of sunitinib in deeper tissues may be achievable through (i) two-photon excitation or (ii) its conjugation with another photosensitizer (see Figure 6). A marked increase in light transmission through tissue can be achieved when using light at 800 nm as compared with 400 nm.^17^ Exciting sunitinib by near-infrared light through a two-photon strategy may solve the tissue penetration issue. Alternatively, other methods can also be used to increase the wavelength of excitation, such as using a chromophore capable of two-photon absorption as a ‘photon harvester', which could then transfer the appropriate energy to sunitinib and excite it. On the other hand, it may be questioned why patients on sunitinib do not have problems with exposure to sunlight. The answer to this is the fact that the excitation wavelength of sunitinib, being in the range of 420 nm, is only minutely represented in the solar spectrum. In addition, most of it is filtered away by the atmosphere. Another strategy could be conjugation of sunitinib with the photosensitizers known to undergo the lysosomal internalization process and characterized by longer, more appropriate light spectra (infrared spectrum region) for excitation.

The lysosomal sequestration of sunitinib, which is a result of its weak basic feature, and its accumulation, due to the protonation of the molecule inside the lysosome, is not unique to sunitinib. The accumulation of other compounds in lysosomes and endocytic vesicles has already been described^8, 18, 19, 20, 21^ and it has recently been suggested to involve the activity of *P*-glycoprotein.^22^ This general response of cells to store toxic compounds in lysosomes suggests a mechanism to protect other cell organelles and processes. The importance of the vascular compartment in a tumor, or in any other tissue, may therefore be reflected by the large number of lysosomes and endocytic vesicles in endothelial cells. Importantly, photodestruction of the lysosomes resulted in a concomitant release of sunitinib, eventually resulting in cell death. This cell death can be caused by interaction with the primary targets, for example, the vascular endothelial growth factor (VEGF)/platelet-derived growth factor (PDGF) receptors, or alternatively, through a toxic side effect induced by the sudden high concentrations of drug in the cytoplasm. In this context, the recent finding of Ellegaard *et al.*^23^ may be relevant. These authors have shown that sunitinib inhibits acid sphingomyelinase to destabilize lysosomes, a mechanism that may be instrumental in the cytotoxic effects observed. Through these effects, photoactivation of sequestered sunitinib may additionally provide a means to target drug-resistant sub-populations of cells, as it has been shown that there is a significant increase in the accumulation of lysosomally sequestered sunitinib in sunitinib-resistant cell cultures as compared with non-resistant parent cell cultures 24 h after incubation with sunitinib.^7^

In the field of photodynamic therapy, several photosensitizers have been shown to exert activity through their accumulation in lysosomes.^9, 24, 25^ Photosensitizers are characterized by their spectral properties and their features to be excitable by light, to generate ROS, such as singlet oxygen or oxidizing radicals, and to exert a cytotoxic activity.^11^ Sunitinib was known to be fluorescent, but a photosensitizing capacity has never been demonstrated.

We found that when excited by light, sunitinib was capable of generating singlet oxygen molecules. Although this activity was relatively weak compared with clinically used photosensitizers, it seemed sufficient to provoke significant biological effects. It is important to realize that the overall biological effect of a photodynamic process depends not only on the efficiency of the photosensitizer to generate ROS but also on the location of the photosensitizer molecules in relation to critical biological targets. The low yield of ROS, such as short-lived singlet oxygen, could in part be compensated by close proximity of the generated ROS to molecular targets, which increases the probability of their interaction. It should also be noted that in our study, the measurements of ROS and singlet oxygen was performed in solution at clinically relevant doses, concentrations that could significantly deviate from the accumulated dose in the lysosomes. Extraction of sunitinib from human tumors revealed that concentrations can be 10-fold higher, as compared with the serum.^7^ Considering the fact that the lysosomal volume may be less than 1% of the volume of the cell, the concentration of sunitinib in a lysosome may be well over 1000-fold higher than in the serum of a cancer patient. This may explain why a successful treatment was achieved, even with the relatively low generation of singlet oxygen molecules.

Major applications of phototherapy are in superficial tumors mainly in the skin and hollow organs, due to the lack of penetration of sufficient light irradiation in deeper tissues. Applications are also possible in transparent parts of the human body, such as the eye. Such applications are used as the principal treatment for polypoidal choroidal vasculopathy,^26^ certain cases of age-related macular degeneration and several ocular tumors.^27^ Phototherapy is also used for skin cancers, including basal cell carcinoma and squamous cell carcinoma.^28, 29, 30^ The combination of phototherapy and angiogenesis inhibition, either before light exposure to generate a vascular normalization window^31^ or after,^32^ in a single drug suggests a benefit for successful application in patients. Alternatively, the capacity of low-dose sunitinib photoactivation as a means of induced vascular leakage and forced delivery of drugs into tumors may also be considered for therapeutic approaches in the clinic.

## Materials and Methods

### Sunitinib preparation and spectroscopy

Stock solutions of the free form of sunitinib (sunitinib malate, Pfizer Inc., New York, NY, USA) were prepared in 100% DMSO (Sigma-Aldrich). Dilutions were made in 0.9% NaCl under continuous stirring. Visudyne (Novartis Pharma Inc.) was dissolved in 5% glucose and diluted in NaCl. Absorption spectra were recorded with a two-beam Varian Cary UV-Vis-NIR 500 Scan spectrophotometer in 1-cm-long quartz cuvettes (Suprasil, Hellma, Müllheim, Germany) between 350 and 800 nm with an average scan speed of 600 nm/min at 20 °C. The steady-state luminescence spectra were recorded with a luminescence spectrometer (Perkin-Elmer, model LS50B, Waltham, MA, USA).

### Cell culture and cellular localization of sunitinib and image quantification

Primary HUVECs were isolated from umbilical cords and cultured in RPMI-1640 (Life Technologies, Breda, The Netherlands) supplemented with 10% heat-inactivated human and 10% heat-inactivated fetal bovine serum, 2 mM l-glutamin (Life Technologies), 50 mg/ml streptomycin and 50 U/ml penicillin (ICN Biomedicals, Santa Ana, CA, USA) in a 0.2% gelatin-coated tissue culture flasks at 37 °C at 5% CO_2_, cells were seeded at a density of 4 × 10^4^ cells/well in 96-well plates. Cells were incubated with 1 *μ*M sunitinib, washed and the cellular localization of sunitinib was evaluated by fluorescence microscopy. Cells were imaged using differential interference contrast microscopy using an Axio Observer microscope at 405 nm (Carl Zeiss, Oberkochen, Germany). Lysosomal sequestration was confirmed by incubation with 25 nM Lysotracker Red DND-99 for 30 min (Invitrogen, Carlsbad, CA, USA). Imaging was performed using a Leica DMIL inverted fluorescence microscope (model 090-135.002, Leica Microsystems GmbH, Wetzlar, Germany). ImageJ (NIH, Bethesda, MD, USA) was used to quantify the number of lysosomes at various time points after light exposure, based on the sunitinib fluorescence.^33^ See Supplementary Material 1.

### Singlet oxygen and superoxide anion measurements

The quantum yield for singlet oxygen photogeneration by sunitinib was determined relative to that of TAP (absolute yield of singlet oxygen generation is 0.7) by measuring time-resolved luminescence intensity in DMSO (Sigma-Aldrich) or acetonitrile (Merck, Darmstadt, Germany) solutions at 1270 nm after excitation of sunitinib at 355 nm. For more information and photosensitized formation of a superoxide anion, see Supplementary Material 2.

### Sunitinib photoactivation, image acquisition and quantification

The chicken CAM^34^ vasculature was visualized and irradiated with light for the photoactivation of sunitinib using an epi-fluorescence microscope (Nikon AG, Eclipse E 600 FN, Tokyo, Japan) with objectives Plan Apo × 4/0.2 or Plan Fluor × 10/0.3 (Nikon AG). Sunitinib was administered intravenously (20 *μ*l) at a concentration of 2 ng/embryo (0.2 *μ*g/kg of body weight) or 12 *μ*g/embryo (1.2 mg/kg of body weight) into one of the main blood vessels of the CAM. One minute after injection, a vascularized area of the CAM was irradiated (*λ*=420 nm) with a light dose of 34 J/cm^2^ and irradiance of 70 mW/cm^2^, or a dose of 130 J/cm^2^ and an irradiance of 270 mW/cm^2^, for each sunitinib dose, respectively. The irradiated area was delimited by an optical diaphragm at 0.02 cm^2^. Visualization of blood vessels was achieved through fluorescence angiography after i.v. injection of FITC-dextran (20 kDa, 20 *μ*l, 25 mg/ml, (*λ*_ex_=470±20 nm, *λ*_em_>520 nm) Sigma-Aldrich).^35^ Image processing and quantification of the fluorescence angiographies in four concentric areas, 1–4, was achieved using a macro written in ImageJ.^36^

### Sunitinib and light illumination in ovarian carcinoma grown on the CAM

A2780 human ovarian carcinoma cells (purchased from ECACC, Salisbury, UK) were maintained at 37 °C and 5% CO_2_ in RPMI-1640 cell culture medium, supplemented as above. Tumors were implanted onto the CAM on the embryo development day 8 by preparing 25-*μ*l hanging drops containing 1 million A2780 cells.^32^ Vascularized tumors were visible 3 days after implantation, when treatment was started. The tumors were divided into three treatment groups: (i) control (0.1% DMSO in 0.9% NaCl; 100 *μ*l injected i.v.), (ii) i.v. sunitinib (12 *μ*g/embryo; 8.75 mg/kg; assuming an embryo weight of 4.3 g, or 100 *μ*l of 700 *μ*M sunitinib), or (iii) i.v. sunitinib+light (12 *μ*g/embryo; 8.75 mg/kg; drug–light interval 1 min; light dose 40 J; light fluence 34 J/cm^2^ at *λ*_ex_=420±20 nm; irradiance 270 mW/cm^2^).

### Colo-26 tumor-bearing mice treatment

Balb/c mice (6–8 weeks old, female, 20 g, Charles River, Orleans, France, approved by the Committee for Animal Experiments for the Canton Vaud) were injected intradermally with 0.5 × 10^6^ Colo-26 cells (purchased from CLS (Eppelheim, Germany) and cultured in RPMI-1640 supplemented with 10% heat-inactivated fetal calf serum and antibiotics) into both flanks. When tumors were 5–6 mm in diameter, mice received 40 mg/kg (0.1 ml) sunitinib i.p., as this dose has been previously reported.^7^ After 3 h, mice were anaesthetized with 2.5% isoflurane, and light treatment was performed using an Oxxius laser (Oxxius SA, Lannion, France) coupled to an optical fiber equipped with a frontal light distributor (Medlight SA, Ecublens, Switzerland) at 405 nm. The applied light fluence of 35 J/cm^2^ was delivered at an irradiance of 100 mW/cm^2^. This treatment was repeated daily for 8 consecutive days, and tumors were measured daily. On the 9th day, mice were killed and tumors were resected.

### Immunohistochemistry

CAM tumors were resected, fixed in zinc fixative solution,^37^ and stained with primary antibody for CD31 (Dianova, DIA-310, Hamburg, Germany).^32^ Murine tumors were stained with primary antibody (Dianova, Anti-Murine CD31 Antibody Clone SZ31). Then CD31 in combination with an indirect alkaline phosphatase method was performed for 1h according to standard procedures (Dianova).

### Statistical analysis

*In vitro* data were analyzed for statistical significance using the unpaired two-tailed *t*-test. Tumor growth curves were analyzed based on the two-way ANOVA or two-tailed *t*-test. Significance was considered at *P<0.05 or **P<0.01.

## Abbreviations

CAM: chicken chorioallantoic membrane
GIST: gastrointestinal stromal tumor
HUVEC: human umbilical vein endothelial cell
PDGF: platelet-derived growth factor
RCC: renal cell carcinoma
ROS: reactive oxygen species
TAP: tetraanilinoporphyrin
VEGF: vascular endothelial growth factor
