# Supplementary material for: Photoactivation of lysosomally sequestered sunitinib after angiostatic treatment causes vascular occlusion and enhances tumor growth inhibition
Source: Cell Death Dis. 2015 Feb 12;6(2):e1641–. doi: 10.1038/cddis.2015.4 (PMC4669819; doi:10.1038/cddis.2015.4)
Supplement: Supplementary Material [file cddis20154x1.doc]

**Supplemental Material**

**Supplemental Material 1.** Lysosome quantification.

ImageJ was used to quantify the number of lysosomes at various time points after light exposure based on the sunitinib fluorescence. Briefly, each cell was analyzed independently by removing background fluorescence (subtracting pixel value from entire image) and selecting a threshold to isolate the highly fluorescent unlysed lysosomes. Images were converted to binary, the watershed function was used to separate touching particles and the open function was used to smooth and remove isolated pixel. Finally, the number of particles was quantified using the ‘analyze particles’ command.

**Supplemental Material 2.** *Singlet Oxygen and Superoxide Anion Measurements*

Solutions of sunitinib in a 1 cm long quartz fluorescence cuvette (QA-1000, Hellma, Müllheim, Germany) were excited by pulses generated using a microchip Nd:YAG laser (Pulselas-P-1064-FC, Alphalas GmbH, Goettingen, Germany) operating at 2-10 kHz repetition rate. To filter out the first and second harmonics of the YAG laser radiation, a 50 cm-length water filter and dichroic mirrors (BK7 series, Eksma Optics, Vilnius, Lithuania) were used. The near-infrared luminescence was measured perpendicularly to the excitation beam in a photon-counting mode [^1^](#_ENREF_1) using a thermoelectric cooled NIR PMT module (H10330-45, Hamamatsu, Japan) equipped with a 1100 nm cut-on filter and an additional dichroic narrow-band filter~~s~~ NBP-1275-2 - 1275 nm NDC Infrared engineering ltd, Bates Road, Maldon, Essex UK). Data were collected using a computer-mounted PCI-board multichannel scaler (NanoHarp 250, PicoQuant GmbH, Berlin, Germany). Data collection was synchronized with laser pulses using an ultrafast photodiode (UGP-300-SP, Alphalas GmbH, Goettingen, Germany) as a trigger. Data analysis, including first order luminescence decay fitted by the Levenberg-Marquardt algorithm, was done by custom-written software. Each experimental point consists of fifteen thousand accumulated acquisitions. The data gathering was performed in a single photon counting mode with 15.000 counts per one measurement point during 10 sec.

Superoxide anion was detected as previously described [^2^](#_ENREF_2). Briefly, 5,5-dimethyl-1-pyrroline N-oxide (DMPO; 0.1 M) (Sigma-Aldrich Chemie GmbH, Steinheim, Germany) was used as a spin trap for the detection of superoxide anion in the solution of the studied compounds in DMSO/H_2_O mixture (3:1). Samples in a 0.25 cm quartz cuvette were irradiated within the resonant cavity of a Bruker EMEX-AA EPR spectrometer (Bruker BioSpin, Ettlingen, Germany) through an optical window with 400-490 nm light derived from a Cermax PE300CE-13FM 300W xenon lamp in air-cooled housing (Perkin Elmer, USA) equipped with a combination of cut-off filter, rejecting irradiation below 390 nm (400FG03 – 50S, Oriel, Newport Corp., Franklin, MA, USA), a dielectric heat reflecting hot mirror, (740nm SIR, Prazisions Glas & Optik GmbH, Iserlohn, Germany) and a blue band-pass filter (370 – 512nm blue additive dichroic filter 505FD64-25, Andover Corporation, Salem, USA). The relevant concentration of the DMPO-spin adduct was monitored continuously by sitting on the low field hyperfine line of the spin adduct EPR spectrum. Relative yields of the photoformation of superoxide anion by the studied compounds in the presence and absence NADH, used as an auxiliary electron donor, were determined by comparing rates of the accumulation of DMPO-OOH determined for the compounds and for riboflavin without EDTA, run under similar experimental conditions.

**Supplemental Material 3.** ROS measurement using EPR-spin trapping

For measurement of ROS we observed the photogeneration of superoxide anion by sunitinib in a mixture of DMSO and H_2_O (3:1). A very small EPR signal of the DMPO spin adduct with superoxide anion could be detected in irradiated samples of sunitinib (below 0.0001, arbitrary units), even in the presence of exogenous electron donors (1 mM NADH). As the yield of the superoxide anion formation was 100-fold lower than that of riboflavin, determined to be 0.01 (not shown), we concluded that photoactivated sunitinib probably did not act significantly through the generation of partially reduced oxygen species.

**Supplemental Material 4.** Tumor growth experiments

Tumor growth on the CAM was measured daily for 4 days following the first treatment. Tumor volumes in CAM and mouse tumors were calculated as follows: volume (mm^3^) = (largest diameter in mm) x (perpendicular diameter in mm)^2^ x 0.52.

**Titles and legends to supplemental figures.**

**Supplemental Figure 1.**


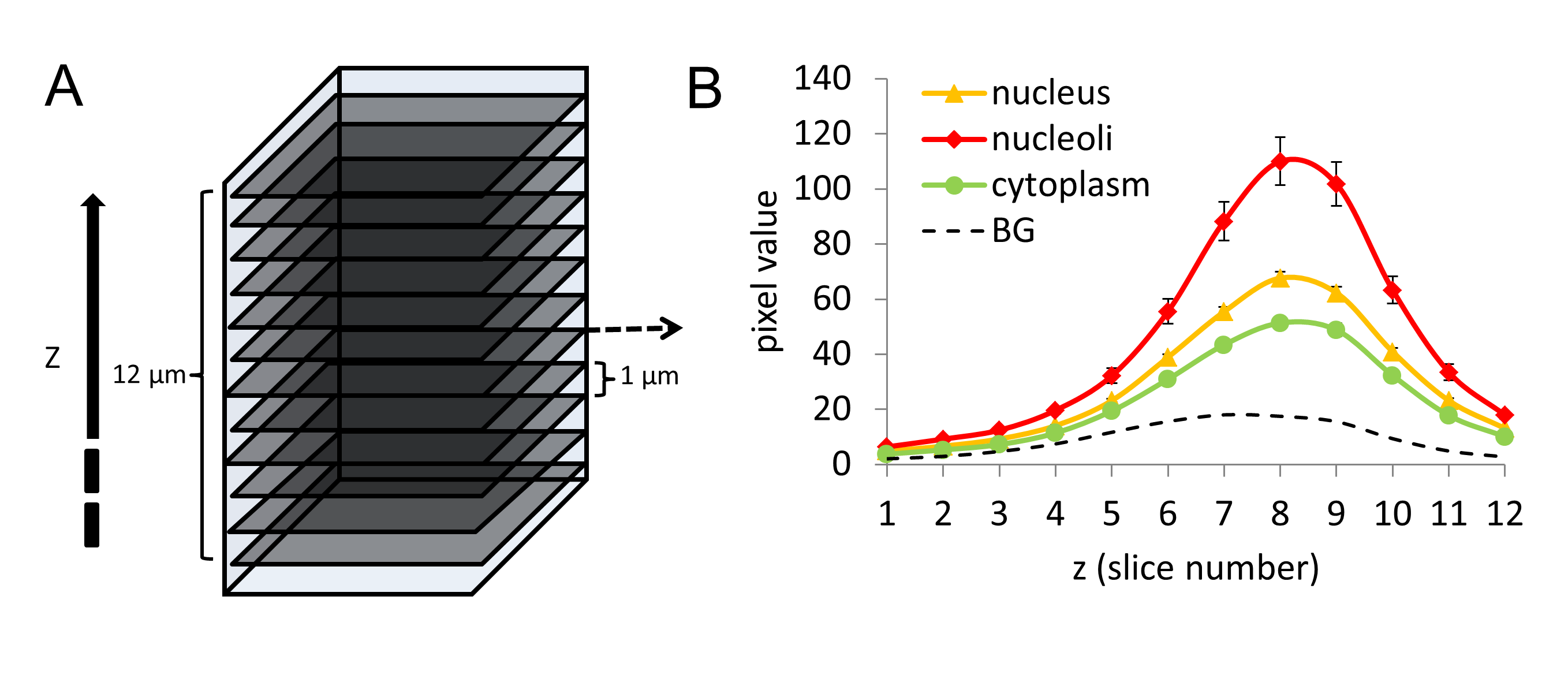


**Suppl. Figure 1.** Confocal microscopy showing nuclear and cytoplasmic localization of sunitinib in RF-24 cells.

A. Z-stack images of confocal fluorescent of RF-24 cells after being incubated for 1 hour in 10 µM sunitinib (λ_ex_ = 405 nm). Images were taken at 20x magnification with a slice thickness of 1 µm. B. Quantification of nuclear, DNA and cytoplasmic fluorescence as a function of the image depth.

**Supplemental Figure 2.**

**
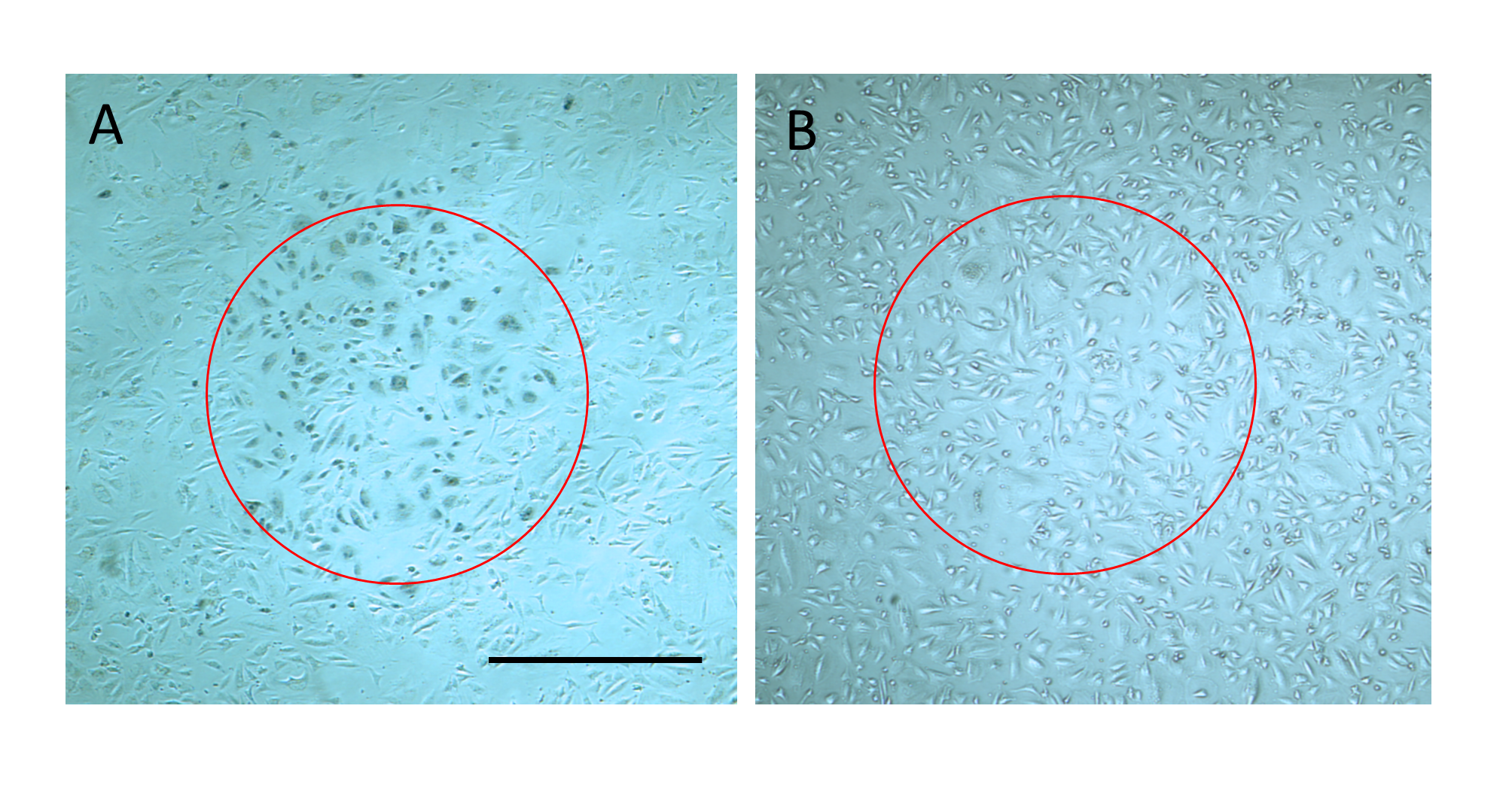
**

**Suppl. Figure 2.** Trypan blue staining to show cell death in HUVEC cells incubated for 1 hour in 1 µM sunitinib and irradiated with light.

A. Sunitinib treatment and irradiation (λ_ex_ = 420 nm) for 60 min. B. HUVEC treated with light only. Light was administered within the area using a diaphragm indicated by the red circle. Cells were stained with trypan blue and imaged 1 hour after light exposure. Bar represents 50 µm.

**Supplemental Figure 3.**


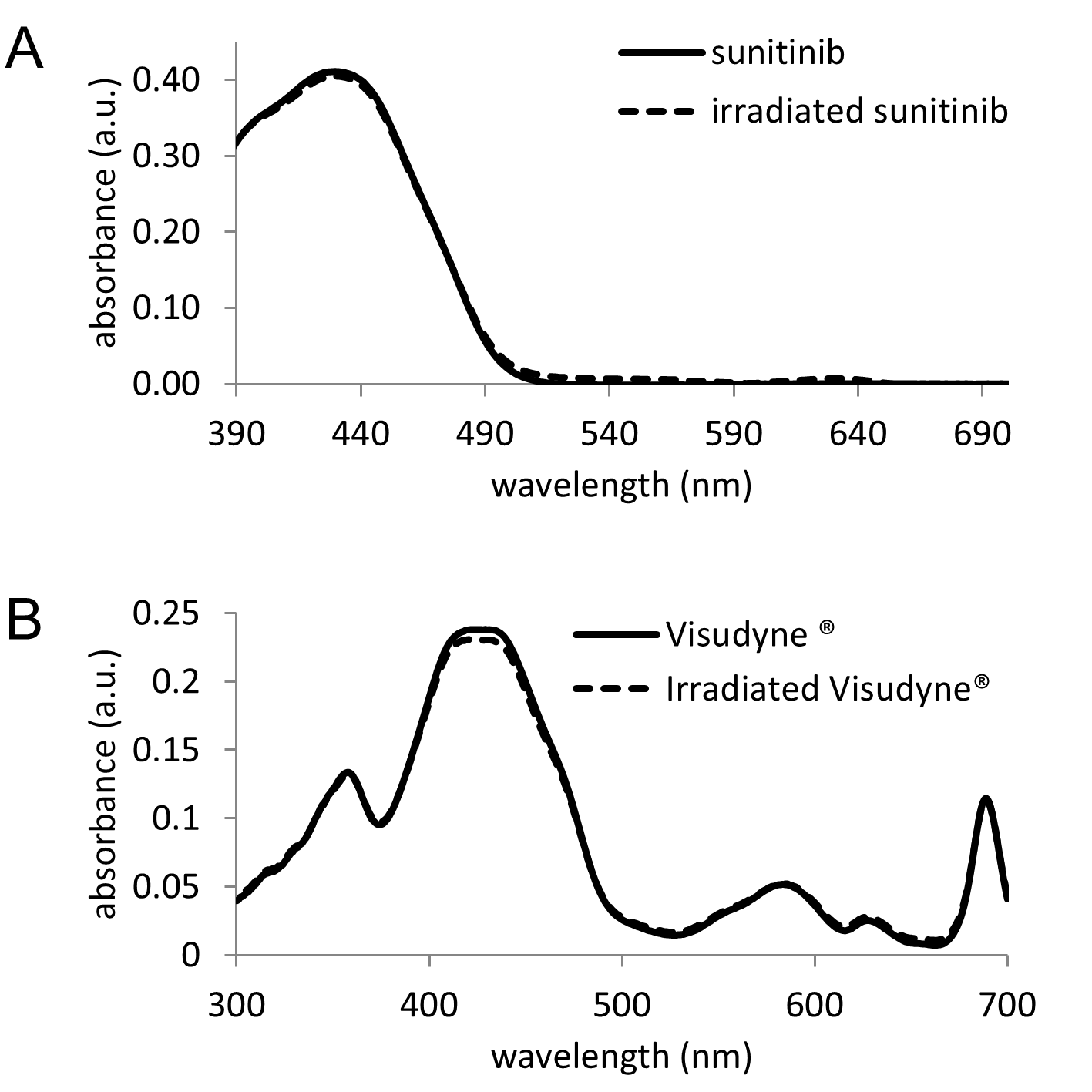


**Suppl. Figure 3.** Effects of irradiation on photosensitizer absorbance.

A. Normalized absorption spectra for sunitinib (100 µM) in 0.02% DMSO in 0.9% NaCl and (B) Visudyne® in 5% glucose at room temperature, both before and after irradiation with 40 J of light at λ_ex_=420 nm.

**Supplemental Figure 4.**


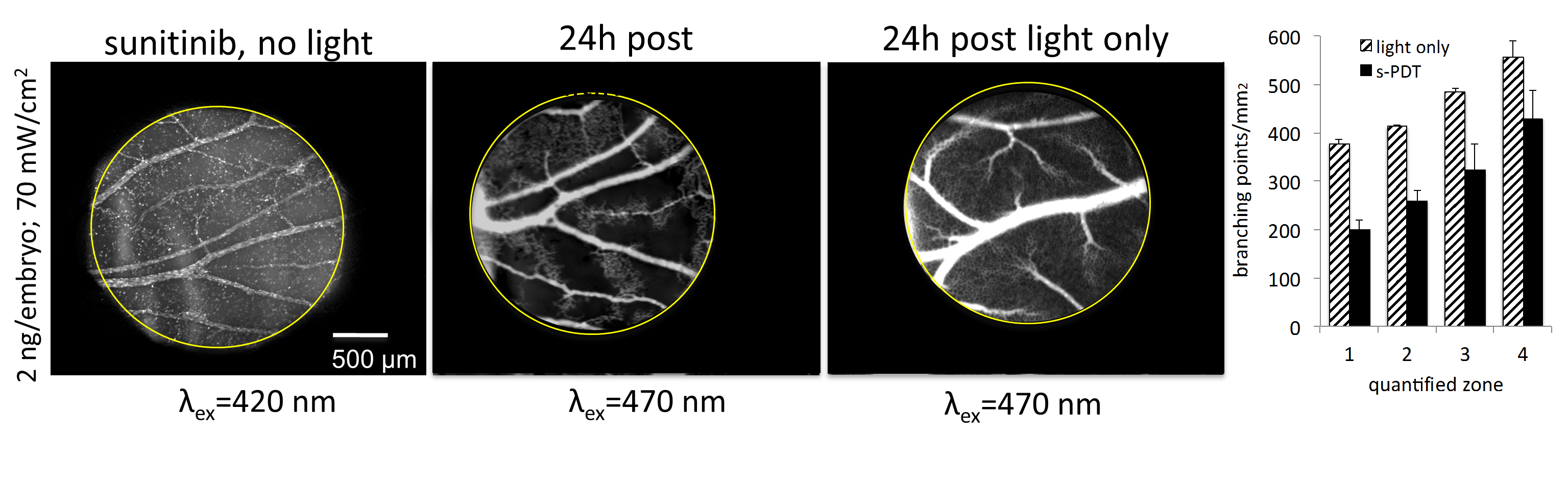


**Suppl. Figure 4.** Vascular effects after suboptimal photoexcitation of sunitinib observed in the CAM model.

A. Sunitinib fluorescence after administration of sunitinib (2 ng/embryo), 1 min prior light exposure (λ_ex_ = 420 nm, 70 mW/cm^2^, left panel). Representative FITC-dextran fluorescence angiography (25 mg/kg, 20 kDa, λ_ex_ = 470 nm, λ_em_ = 520 nm) 24 h after sunitinib treatment and light exposure (middle panel). Representative fluorescence angiography taken 24 h post light only treatment (270 mW/cm^2^, right panel). The bar in A represents 500 µm. B. Quantification of branching points/mm^2^ for areas 1 (**p=0.007), 2 (**p=0.009), 3 (*p=0.019) and 4 (*p=0.029), calculated as shown in Figure 3. Results are expressed as means ± SEM.

**Supplemental References**

1. A. Jimenez-Banzo, X. Ragas, P. Kapusta and S. Nonell, *Photochemical & photobiological sciences : Official journal of the European Photochemistry Association and the European Society for Photobiology*, 2008, 7, 1003-1010.

2. L. Panzella, G. Szewczyk, M. d'Ischia, A. Napolitano and T. Sarna, *Photochemistry and photobiology*, 2010, 86, 757-764.
